# Stages identifying and transcriptome profiling of the floral transition in *Juglans regia*

**DOI:** 10.1038/s41598-019-43582-z

**Published:** 2019-05-08

**Authors:** Shaowen Quan, Jianxin Niu, Li Zhou, Hang Xu, Li Ma, Yang Qin

**Affiliations:** 1 Department of Horticulture, College of Agriculture, Shihezi University, Shihezi, 832003 Xinjiang China; 2 Xinjiang Production and Construction Corps Key Laboratory of Special Fruits and Vegetables Cultivation Physiology and Germplasm Resources Utilization, Shihezi, 832003 Xinjiang China

**Keywords:** RNA sequencing, RNA sequencing, Shoot apical meristem, Shoot apical meristem

## Abstract

Using paraffin sections, the stages of walnut female flower bud differentiation were divided into the predifferentiation period (F_1), initial differentiation period (F_2) and flower primordium differentiation period (F_3). Leaf buds collected at the same stage as F_2 were designated JRL. Transcriptomic profiling was performed, and a total of 132,154 unigenes were obtained with lengths ranging from 201 bp to 16,831 bp. The analysis of differentially expressed genes (DEGs) showed that there were 597, 784 and 532 DEGs in the three combinations F_1vsF_2, F_1vsF_3, and F_2vsF_3, respectively. The comparison F_2vsJRL showed that 374 DEGs were differentially expressed between female buds and leaf buds. Thirty-one DEGs related to flowering time were further used to construct coexpression networks, and CRY2 and NF-YA were identified as core DEGs in flowering time regulation. Eighteen DEGs related to flowering time were subjected to real-time quantitative analysis. Our work provides a foundation for further research on the walnut floral transition and provides new resources for future research on walnut biology and biotechnology.

## Introduction

*Juglans regia* is widely cultivated for its commercially valuable timber and nuts in China and other temperate parts of the world^1^. In 2016, global production of walnuts (in shell) was 3.7 million metric tons, and China was the leading producer worldwide with 41% of total production (FAO, 2017)^2^. In contrast to annual plants, walnut trees require several years before flowering and to bear fruit after seed germination, and little has been reported about the mechanism of the floral transition in walnut.

Many studies have been performed on the floral transition in *Arabidopsis thaliana* and other plants^3–12^. Six genetic pathways (including the photoperiod, vernalization, temperature, gibberellin, autonomy and age pathways) and several flowering integrator genes (including *FLOWERING LOCUS T* (*FT*), *LEAFY* (*LFY*), and *SUPPRESSOR OF OVEREXPRESSION OF CONSTANS* 1 (*SOC1*)) have been shown to regulate flowering time in *Arabidopsis thaliana*^3,6–8,12–18^. However, few flowering time genes have been identified in walnut.

In this study, sections of paraffin-embedded tissues were used to observe the morphogenesis of floral buds in *Juglans regia*, and a high-throughput sequencing platform was used to sequence cDNA libraries at each stage of the floral transition. We investigated the differentially expressed genes (DEGs) involved in the developmental process of the walnut floral transition, and our results demonstrated that DEGs between the stages before and during the floral transition played a key role in regulating the floral transition. These results provide essential information on the genes and pathways involved in floral transition development in the walnut and may help in further researching the molecular mechanism of walnut flowering.

## Results

### Morphological characteristics of walnut floral transition

**Figure 1:**
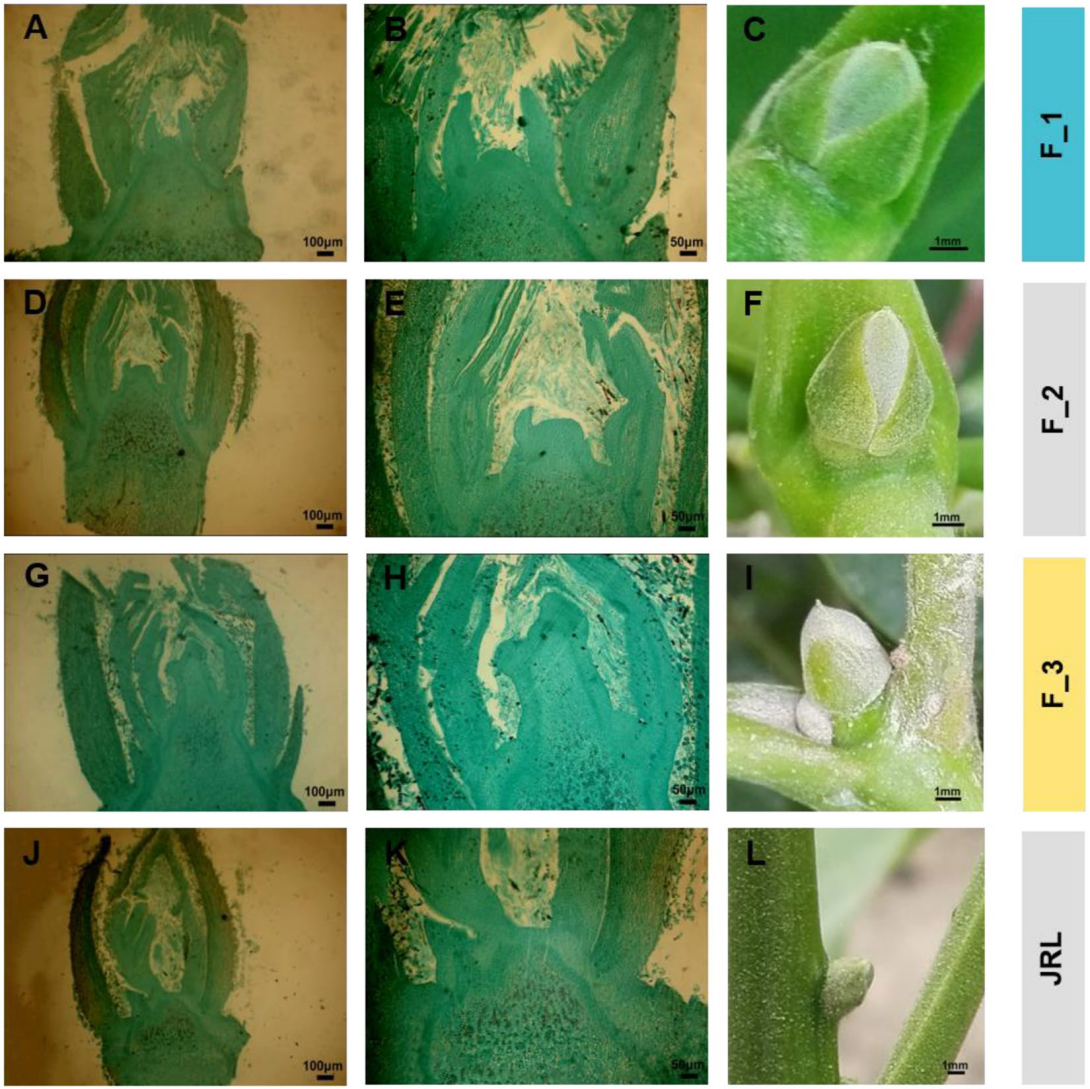
Morphological characteristics of walnut female flower buds. (**A**–**C**) show the female flower buds in the predifferentiation period identified in the undifferentiated stage (F_1); (**D**–**F**) show the female flower buds in the initial differentiation period (F_2); (**G**–**I**) show the female flower buds identified at the flower primordium differentiation period (F_3); (**J**–**L**) show the leaf buds (JRL) collected at the same stage of F_2. (**A**,**D**,**G**,**J**) are images of paraffin sections with a 100 μm scale bar; (**B**,**E**,**H**,**K**) are images of paraffin sections with a 50 μm scale bar; (**C**,**F**,**I**,**L**) show the external morphology of the buds at different stages with a 1 mm scale bar.

Morphological differentiation of female flower buds in walnut was continuously observed by paraffin section. We divided walnut female flower bud differentiation into stages as follows: F_1 (predifferentiation period), F_2 (initial differentiation period) and F_3 (flower primordium differentiation period). In the predifferentiation period (F_1), the growth points of the flower bud appeared flat, and the bud scales appeared light green. In the initial differentiation period (F_2), the flower stalk primordium was raised, and the bud scales were yellow-green. In the flower primordium differentiation period (F_3), the primordium of the flower stalk continued to extend, the pistil primordium began to appear, and the bud scales appeared yellow-green (Fig. 1). The leaf buds (JRL) were collected at the same stage as F_2.

### General analysis of walnut transcriptome data

#### Sequencing, splicing, and annotation of transcriptome data

**Figure 2:**
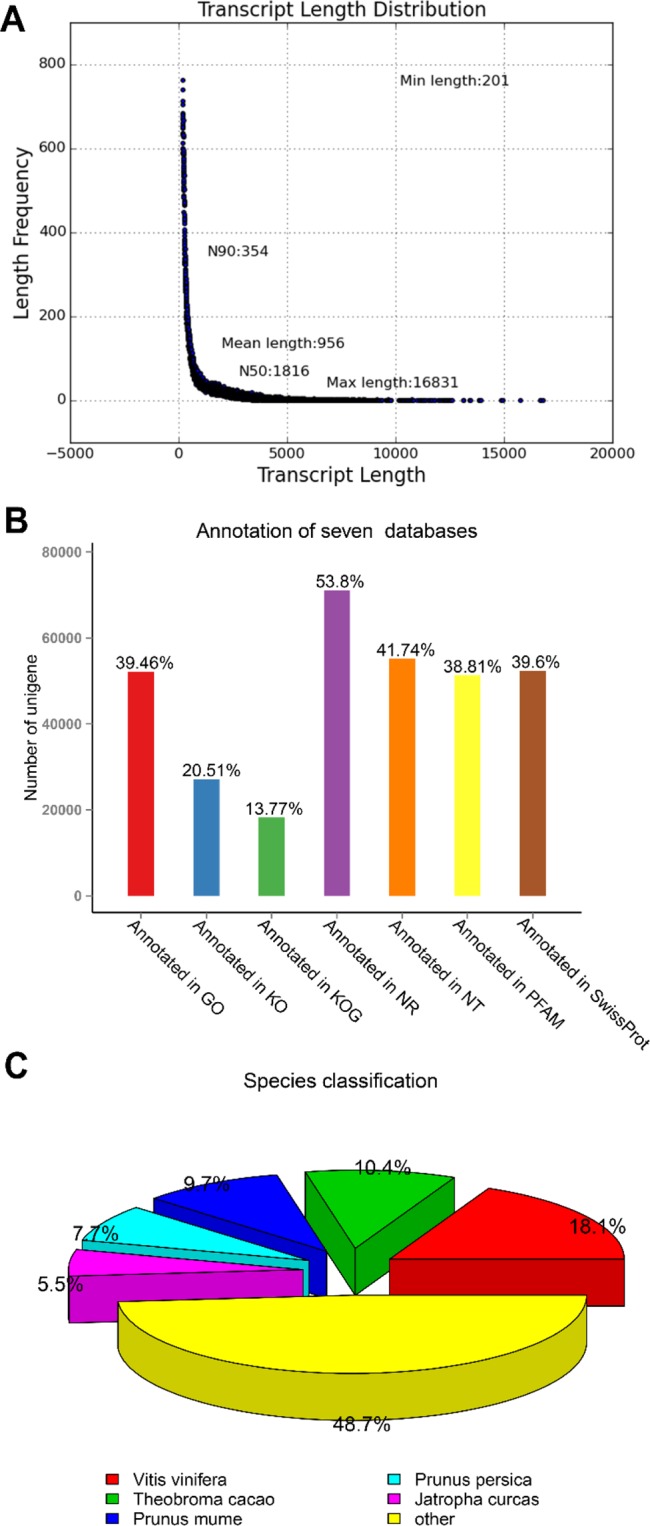
Analysis of the transcriptome data. (**A**) length distribution of transcripts; (**B**) transcriptome data annotated in seven databases; (**C**) the species classification of the walnut transcriptome data annotated by the NR database.

A total of 194,926,208 raw reads were obtained through the sequencing of the transcript libraries (Table 1). By eliminating chimeric and low-quality reads, 189,041,550 clean reads were finally screened, and 132,154 unigenes were obtained with a mean length of 956 bp; the lengths of the unigenes ranged from 201 bp to 16831 bp (Fig. 2A).

**Table 1:** Quality of data output.

Sample	Raw reads	Clean reads	Clean bases	Error (%)	Q20 (%)	Q30 (%)	GC (%)
F_1	57270794	56064740	8.41G	0.02	96.86	92.20	44.83
F_2	42190694	40686164	6.1G	0.03	94.41	86.66	44.92
F_3	48518386	46937334	7.04G	0.03	94.79	87.59	45.15
JRL	46946334	45353312	6.8G	0.03	94.63	87.12	44.94

All unigenes were compared with 7 databases (NR, NT, KEGG, Swiss-Prot, PFAM, GO, and KOG/COG) with an E value cutoff of e-5 for homology. Then, the unigenes were annotated according to the results: 39.46% of unigenes were annotated by the GO database, 20.51% of unigenes were annotated by the KO database, 13.77% of unigenes were annotated by the KOG database, 53.8% of unigenes were annotated by the NR database, 41.74% of unigenes were annotated by the NT database, 38.81% of unigenes were annotated by the PFAM database, and 39.6% of unigenes were annotated by the Swiss-Prot database. In total, 78,718 unigenes of the 132,154 total unigenes were annotated to at least one database, accounting for 59.56% of all unigenes, and 11,165 unigenes were annotated by all seven major databases, accounting for 8.44% of all unigenes (Fig. 2B).

The annotation results of the NR database show that 11372 unigenes (18.1% of the total annotated unigenes) were compared to *Vitis vinifera*, 6522 unigenes (10.4% of the total annotated unigenes) were compared to *Prunus mume*, 6141 unigenes (9.7% of the total annotated unigenes) were compared to *Prunus persica*, 4834 unigenes (7.7% of the total annotated unigenes) were compared to *Jatropha curcas*, 3457 unigenes (5.5% of the total annotated unigenes) were compared to *Citrus sinensis*, and the remaining 48.7% unigenes were compared to other species (Fig. 2C).

#### GO enrichment

**Figure 3:**
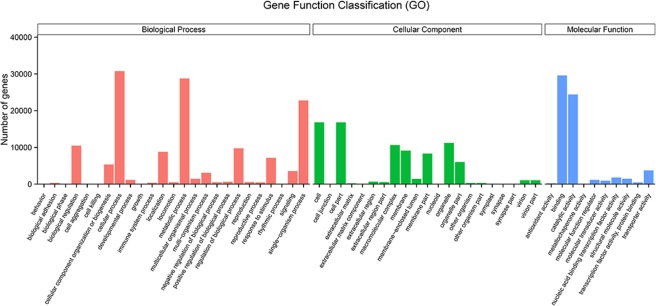
Gene numbers of the enriched GO terms.

**Figure 4:**
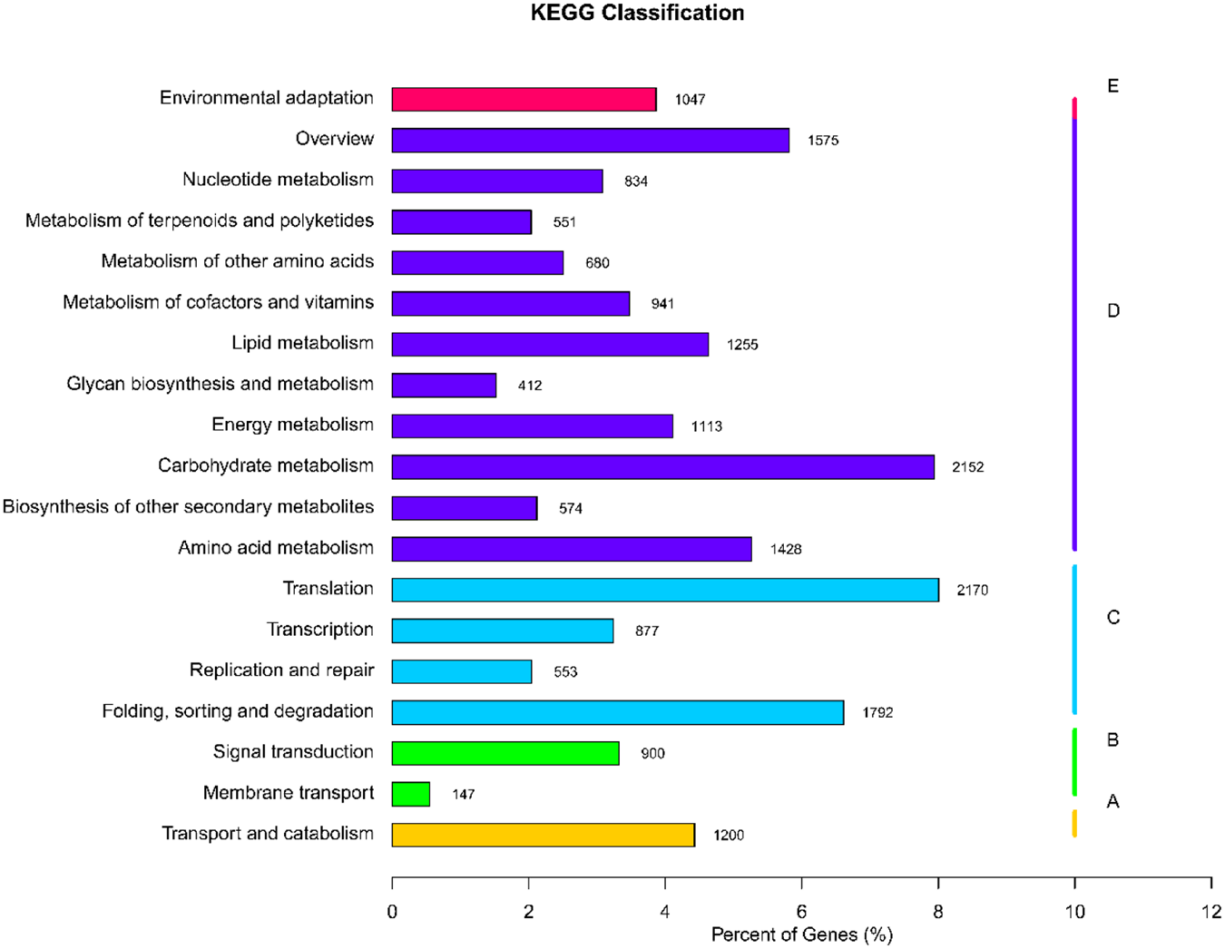
KEGG pathway analysis of the transcriptome data. The abscissa is the ratio of the single pathway gene number to the total annotated gene number, and the ordinate is the name of the KEGG metabolic pathway. We divided the involved genes into 5 branches: (**A**) Cellular Processes; (**B**) Environmental Information Processing; (**C**) Genetic Information Processing; (**D**) Metabolism; (**E**) Organismal Systems.

The gene functional classifications were identified in a GO enrichment analysis based on the 52,157 unigenes annotated in the GO database. In the biological process category, cellular process, metabolic process, and single organism process were highly represented. In the cellular component category, significantly enriched genes were associated with cell, cell part, and organelle. In the molecular function category, GO terms related to binding, catalytic activity and transporter activity were significantly enriched (Fig. 3). *KEGG pathway enrichment*. KEGG pathway enrichment analysis showed that transport and catabolism, signal transduction, transition, carbohydrate metabolism and environmental adaptation had the most unigenes in classifications of Metabolism, Genetic Information Processing, Environmental Information Processing, Cellular Processes and Organismal Systems, respectively (Fig. 4). In particular, Environmental Information Processing and Organismal Systems, which included many subterms associated with flowering, such as the pathways of plant hormone signal transduction (ko04075) and Circadian rhythm-plant (ko04712), were enriched (Table S1).

#### Transcription factor (TF) enrichment analysis

**Figure 5:**
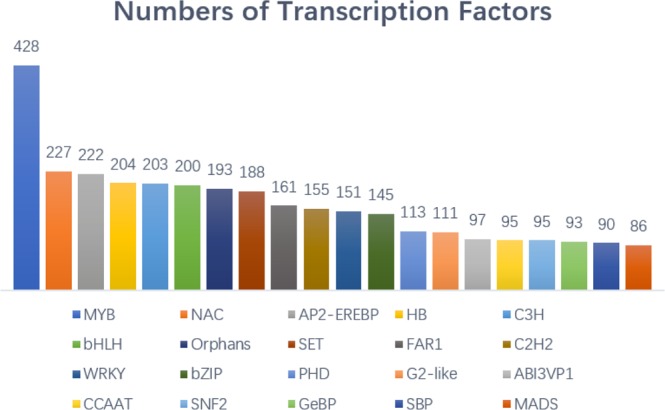
Transcription factor enrichment analysis of the walnut transcriptome data.

TFs function alone or with other proteins in complexes by promoting or blocking the recruitment of RNA polymerase to specific genes^19–21^. Some TFs have been shown to play key roles in plant flowering^22–24^. Among the 132,154 unigenes, 4,835 of the unigenes (approximately 3.7%) were annotated as TFs, and they fell into diverse categories covering nearly all TF families, with MYB, NAC, and AP2-EREBP being the most highly represented (Fig. 5).

### DEGs analysis of walnut transcriptome data

#### Identification of DEGs

The four databases from F_1, F_2, F_3, and JRL were used for paired comparisons, and four combinations (F_1vsF_2, F_1vsF_3, F_2vsF_3, F_2vsJRL) were chosen and analyzed.

**Figure 6:**
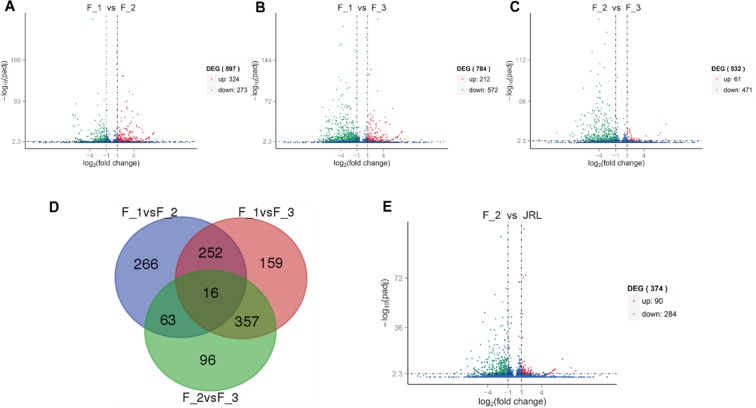
Numbers of DEGs in F_1, F_2, F_3 and JRL. (**A**–**C**,**E**) volcano plot of the DEGs in F_1vsF_2, F_1vsF_3, F_2vsF_3 and F_2vsJRL. The abscissa represents the multiple expression of genes in different samples. The ordinate represents the statistical significance of the change in gene expression. The scattered points in the map represent the genes, and genes without significant differences are presented as blue dots. Upregulated genes and downregulated genes with significant differences are presented as red and green dots, respectively; (**D**) Venn diagram of F_1vsF_2, F_1vsF_3 and F_2vsF_3.

**Figure 7:**
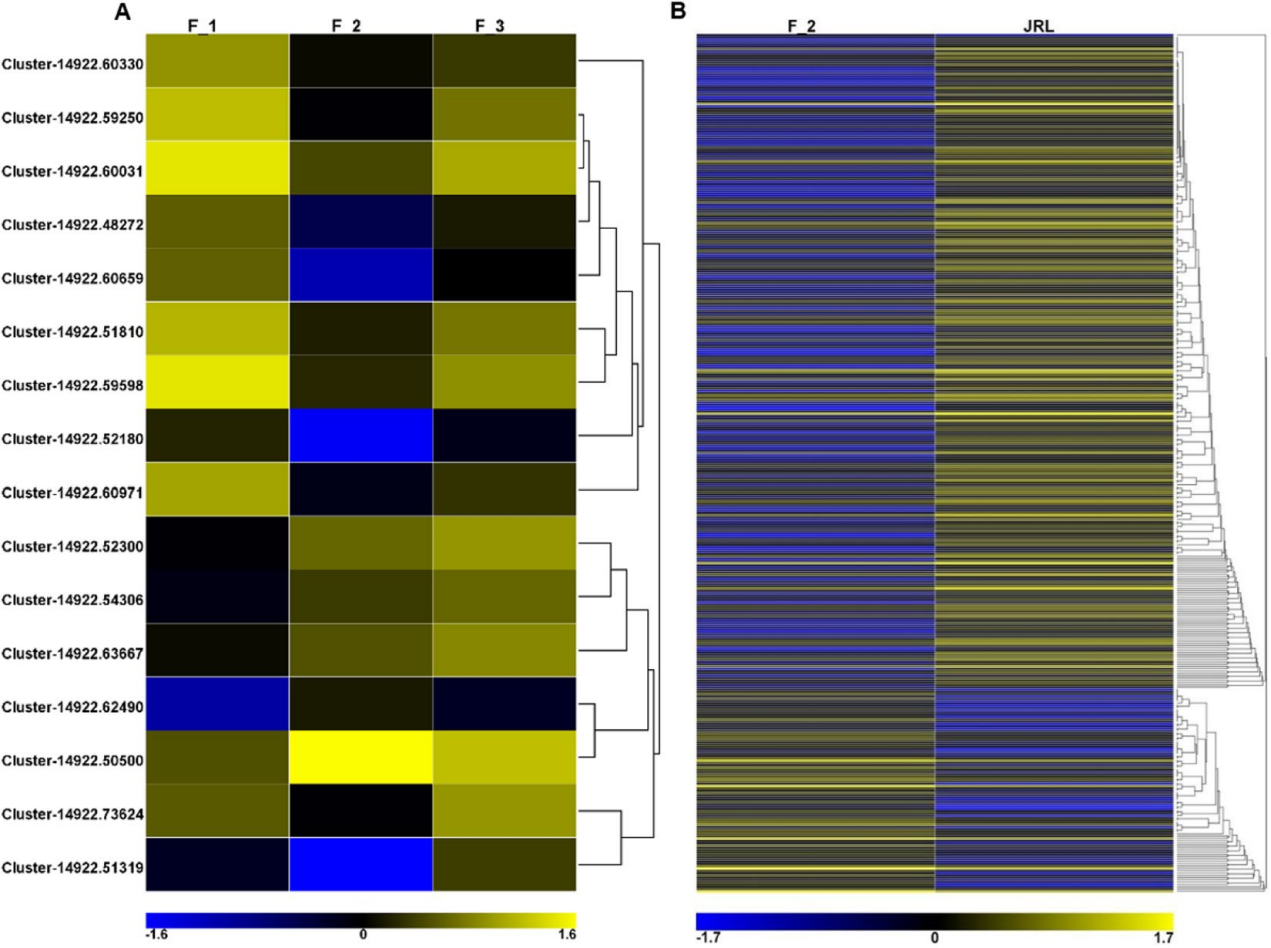
(**A**) Heatmap of 16 DEGs expressed differently in three stages of female flower buds (F_1, F_2, and F_3); (**B**) Heatmap of DEGs between female flower buds (F_2) and leaf buds (JRL).

The three combinations of female flower bud developmental stages (F_1vsF_2, F_1vsF_3, F_2vsF_3) had 597 DEGs, 784 DEGs and 532 DEGs, respectively. In addition, 16 DEGs were shared by all three combinations, which means they were expressed differently in the three periods of female flower buds (Fig. 6). The expression levels of DEGs between F_1, F_2 and F_3 were plotted in a heatmap (Fig. 7A).

In addition, the female flower buds at the beginning of differentiation (F_2) and the leaf buds of the same stage (JRL) were used for comparison (F_2vsJRL). The results showed that 374 DEGs were expressed differently in female buds (F_2) and leaf buds (JRL), of which 90 unigenes in the female flower buds (F_2) showed a higher expression level than that in the leaf buds (JRL), and the other 284 unigenes showed the opposite trend (Fig. 6E). The expression levels of 374 DEGs between female buds (F_2) and leaf buds (JRL) were plotted in a heatmap (Fig. 7B).

#### GO functional analysis of DEGs

**Figure 8:**
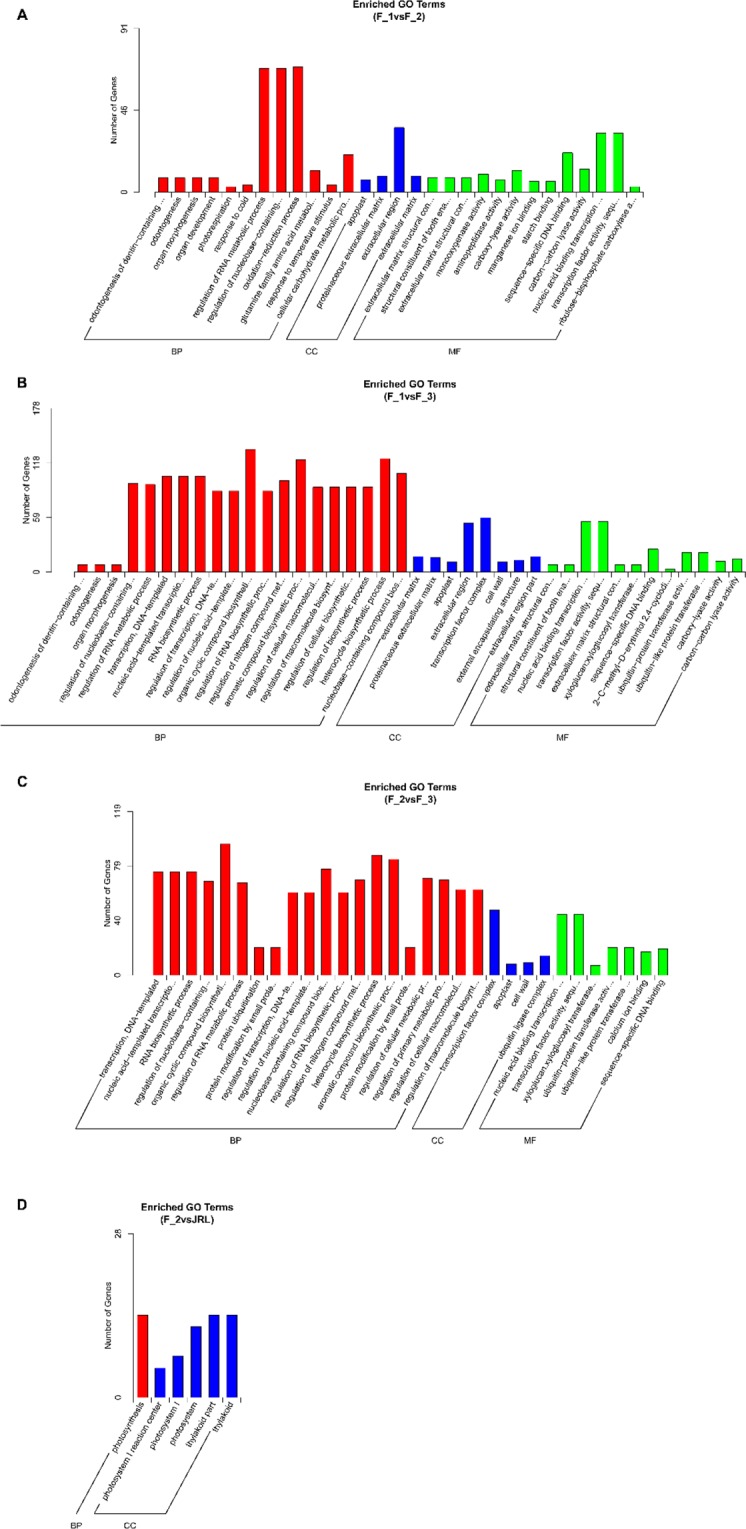
(**A**–**D**) represent the enriched GO terms of the DEGs in F_1vsF_2, F_1vsF_3, F_2vsF_3 and F_2vsJRL, respectively. The upregulated GO terms are shown with red bars, and the downregulated GO terms are shown with blue bars.

We conducted GO functional analysis with the upregulated and downregulated DEGs of F_1vsF_2, F_1vsF_3, and F_2vsF_3, and the results are shown in Fig. 8. The results indicated that the DEGs of the three stages of female flower bud differentiation had functions enriched in the regulation of RNA metabolic process (GO:0051252, BP); regulation of nucleobase-containing compound metabolic process (GO:0019219, BP); apoplast (GO:0048046, CC); nucleic acid binding transcription factor activity (GO:0001071, MF); transcription factor activity, sequence-specific DNA binding (GO:0003700, MF); and sequence-specific DNA binding (GO:0043565, MF). In addition, the GO functions of the DEGs between female flower buds (F_2) and leaf buds (JRL) were enriched in photosynthesis (GO:0015979, BP), photosystem I reaction center (GO:0009538, CC), photosystem I (GO:0009522, CC), photosystem (GO:0009521, CC), and thylakoid part (GO:0044436, CC).

**Figure 9:**
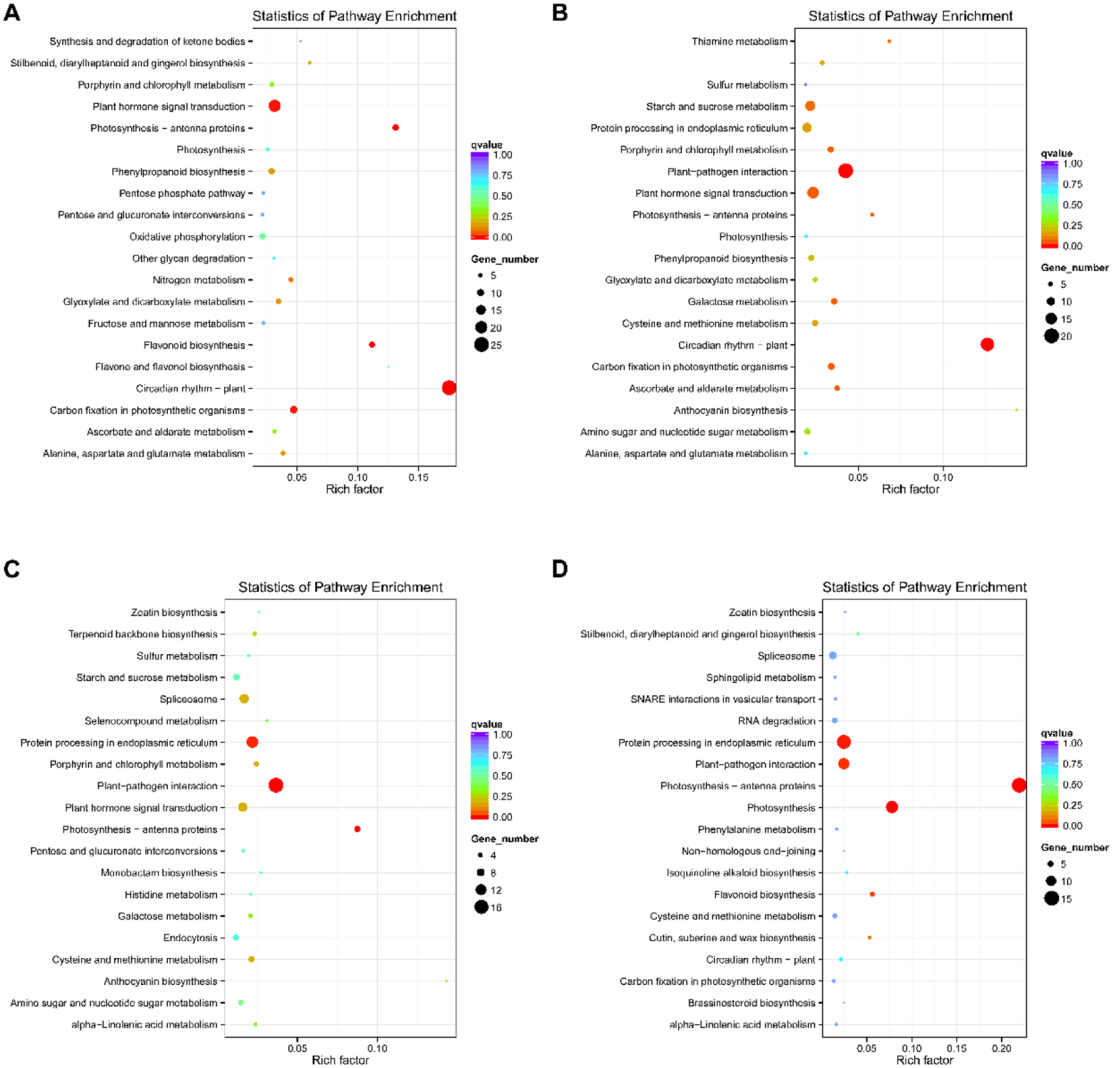
(**A**–**D**) represent scatterplots of the top 20 enriched KEGG pathways of the DEGs in F_1vsF_2, F_1vsF_3, F_2vsF_3 and F_2vsJRL, respectively. The vertical axis represents the pathway names, the horizontal axis represents the rich factors corresponding to the pathways, the size of the q value is represented by the color of the dots, and the number of differentially expressed genes in each pathway is expressed by the size of the dots.

KEGG pathway analysis of DEGs: KEGG pathway analysis of the DEGs of F_1VSF_2, F_1VSF_3, F_2VSF_3 showed that most pathways were associated with flowering, and three pathways (Photosynthesis-antenna proteins, Plant hormone signal transduction, Porphyrin and chlorophyll metabolism) were shared by the three combinations. In addition, the pathway enrichment analysis of female flower buds (F_2) and leaf buds (JRL) also included many flowering-related pathways (Fig. 9).

Analysis of DEGs related to flowering time: The flowering time genes that induce plant flowering are usually attributed to the photoperiod, vernalization, autonomous, GA, and age-regulated pathways, as well as the integrated factors of the intersection of these pathways^3,6,13,25^.

Based on previous studies, we summarized the important flowering time genes in the model plant *Arabidopsis thaliana*, and these genes were used as query genes (Table 2). Finally, we screened 31 DEGs associated with flowering time in walnut, and eighteen of the flowering time DEGs were chosen for qRT-PCR.

**Table 2:** Important genes involved in flowering time pathways. The number of walnut DEGs is shown after each gene name.

Flowering Pathways	Involved Genes (Number)
Photoperiod	*CCA1* (*0*), *CDFs* (*2*), *CO* (0), *COP1* (*0*), *CRY2* (*1*), *DNF* (*0*), *ELF3* (*0*), *ELF4* (*0*), *FD* (*0*), *FKF*1 (*1*), *FLD* (*0*), *FLK* (*0*), *GI* (*2*), *LHY* (*2*), *NF-Ys* (*3*), *PHYA* (*0*), *PHYB* (*0*), *PHR*2 (*2*), *PIF3* (*1*), *PRRs* (*8*), *SMZ* (*0*), *SNZ* (*0*), *SPA* (*0*), *TEM1* (*2*),*TEM2* (*0*), *TOC1* (*0*)
Vernalization	*FLC* (*0*), *FRI* (*0*), *LHP1* (*0*), *VIL2* (*1*), *VIN3* (*0*), *VRN2* (*0*), *VRN1* (*0*), *VRN2* (*0*)
Autonomous	*FCA* (*0*), *FLC* (*0*), *FLD* (*0*), *FLK* (*0*), *FLM* (*0*), *FPA* (*0*), *FVE* (*0*), *FY* (*0*), *LD* (*0*), *SVP* (*0*), *REF6* (*0*)
Ambient temperatures	*ARP6* (*0*), *HOS1* (*0*), *SVP* (*0*), *HVP* (*0*), *miR156* (*0*), *SPL3* (0)
GA	*GA1* (*0*), *GAI* (*0*), *GID* (*1*), *GNC* (*0*), *GNL* (*0*), *RGA* (*0*), *RGL1* (*0*), *RGL2* (*0*), *RGL3* (*0*), *PY* (*0*), *FUL* (*0*), *RGA* (*0*)
Age regulated	*SPLs* (*0*), *MIR156* (*0*), *MIR172* (*0*), *JMJ18* (*0*), *TPS1* (*3*)
Integrators	*FT* (*0*), *LFY* (*0*), *SOC1* (*0*)
Supplement	*AP1* (*2*)

#### DEGs related to the walnut floral transition

**Figure 10:**
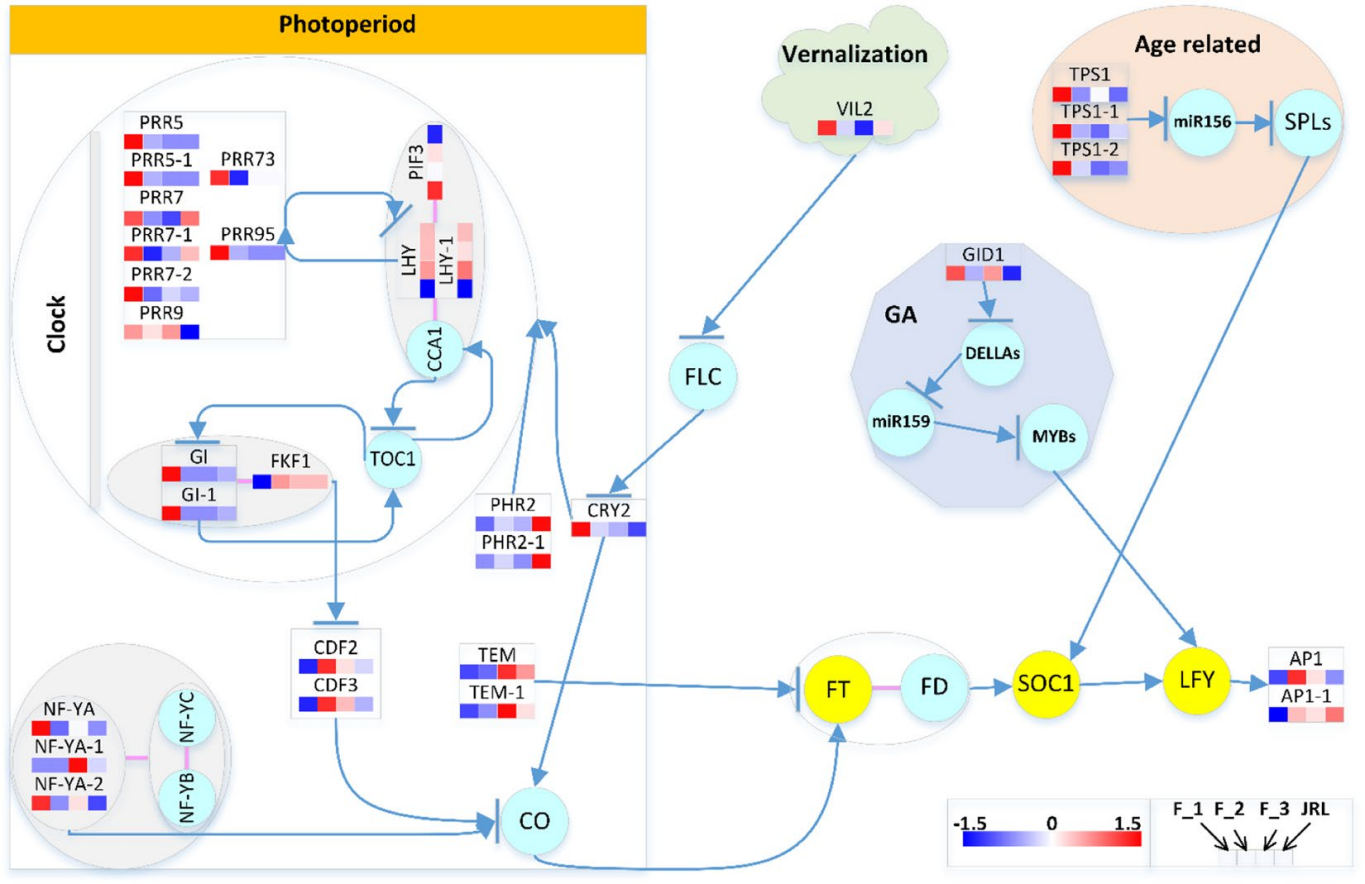
Heatmap of flowering-time-related DEGs. The DEG expression levels are indicated by color code according to the scale. The squares from left to right indicate the samples in F_1, F_2, F_3, and JRL. Genes within the circle were not differentially expressed in our study; circles filled with azure blue indicate normal genes, and circles filled with yellow indicate integral genes. Arrows indicate promotion, and arrows with a bar indicate inhibition. Red lines between genes indicate that these genes can combine to form a complex. The heatmap was drawn with HEMI according to the DEG expression, and the flow chart was drawn with Visio.

A total of 31 unigenes were involved in the photoperiod (circadian rhythm), vernalization pathway, GA, and age-related pathways. To investigate the complex molecular mechanisms underlying floral transition in walnut, we set the 31 flowering time-related DEGs in the flowering time regulation network (Fig. 10).

Most of the DEGs were annotated in the photoperiod pathway: In total, twenty-four DEGs were annotated in this pathway, fifteen DEGs were highly expressed in the predifferentiation period (F_1), including *CRY*2 (1), *FKF1* (1), *GI* (2), *NF-YA* (2), *PIF*3 (1), *PPR5* (2), *PRR7* (3), *PRR9* (1), *PRR73* (1), and *PRR95* (1). *CDF*2 (1), *CDF3* (1), and *LHY* (2) were highly expressed in the initial differentiation period (F_2). *NF-YA* (1) and *TEM1* (2) were highly expressed in the flower primordium differentiation period (F_3). Two *PHR*2 genes had higher expression levels in leaf buds (JRL) than in flower buds (F_2), while *PRR9* exhibited the opposite trend.

In *Arabidopsis thaliana*, *CYCLING DOF FACTOR 2* (*CDF2*) and *CYCLING DOF FACTOR* 3 (*CDF3*) act redundantly to reduce CONSTANS (CO) expression and are degraded by the complex of *GIGANTEA* (*GI*) and *FLAVIN-BINDING KELCH REPEAT F-BOX* 1 (*FKF*1), thus releasing repression of *CO* and *FT* transcription^26–28^. Blue-light photoreceptors such as *PHOTOLYASE-RELATED 2* (*PHR2*) are essential light detectors for the early development of plants and mediate phototropism and the expression of specific genes. Loss of a blue-light photoreceptor in the hy4 mutants of *Arabidopsis thaliana* substantially delayed flowering^29,30^. *CRYPTOCHROME 2* (*CRY2*) can promote the expression of *FT* and is negatively regulated by *FLOWERING LOCUS C* (*FLC*)^31^. The NUCLEAR FACTOR Y (NF-Y) transcription factors (heterotrimeric complexes composed of NF-YA and the dimer of NF-YB/NF-YC) can initiate photoperiod-dependent flowering by cooperatively interacting with CO to drive the expression of *FT*^32^.

*Phytochrome Interacting Factor 3* (*PIF3*) can bind the promoters of *LATE ELONGATED HYPOCOTYL* (*LHY*) and *CIRCADIAN CLOCK ASSOCIATED* 1 (*CCA1*) to form an *in vitro* ternary complex. Additionally, the complex of *CCA1* and *LHY* can bind with *TIMING OF CAB 1* (*TOC1*) to form a feedback loop that is necessary for the circadian clock in *Arabidopsis thaliana*^33^. In addition, the *CCA1* and *LHY* complexes were repressed by the *Pseudo Response Regulators* (*PRRs*) encoded by *TOC1*^6,34^. The activator CO and the repressor TEMPRANILLO (TEM) have a quantitative balance that determines the *FT* transcription level, as it shifts the CO/TEM balance in favor of CO activity, allowing *FT* transcription to reach the threshold level required to trigger flowering^35^.

*In the age-regulated pathway*, three DEGs were annotated as *TREHALOSE-6-PHOSPHATE SYNTHASE 1* (*TPS1*), which is essential for normal vegetative growth and transition to flowering^36,37^. They were differentially expressed between the different flower bud formation stages, and the flower buds showed no difference from the leaf buds in their expression level.

*In the vernalization pathway*, vernalization can cause epigenetic changes in FLC and indirectly promote flower formation by histone modification, and *MADS* AFFECTING *FLOWERING 5* (*MAF5*) is an FLC-related gene^38–43^. *VERNALIZATION INSENSITIVE 3-LIKE* 2 (*VIL2*), which can repress MAF5 and permit more rapid flowering during noninductive photoperiod conditions in *Arabidopsis thaliana*^44^. In this study, *VIL2* was highly expressed in the predifferentiation period (F_1), decreased in the initial differentiation period (F_2), and decreased to the lowest point at the flower primordium differentiation period (F_3). In the leaf buds (JRL), its expression level was higher than that in the flower buds during the same period (F_2).

*In the GA pathway*, GA regulates *LEAFY* (*LFY*) via DELLAs, *miR159* and *MYB*s^45–47^. The combination of gibberellin and GIBBERELLIN-INSENSITIVE DWARF1 (GID1) can combine with the DELLA protein to form a GA-GID1-DELLA trimer, and then the SKP1-CUL1-F-box (SCF) polymer can tag the trimer to induce the ubiquitin 26 S proteasome to degrade the DELLA protein, relieving the inhibitory effect of the DELLA protein on plant growth and producing gibberellin effects^48,49^. In this study, GID1 was highly expressed in the predifferentiation period (F_1), and its expression was downregulated in the initial differentiation period (F_2). In addition, the expression of GID1 was higher in flower buds (F_2) than in leaf buds (JRL).

In addition to the DEGs mentioned above, there are two DEGs annotated as *APETALA1* (*AP1*). In *Arabidopsis thaliana*, *AP1* is required for the floral transition^50^. In this study, the two AP1 genes were nearly undetectable in the predifferentiation period (F_1), and their expression showed a significant upregulation in the initial differentiation period (F_2).

### Coexpression networks

**Figure 11:**
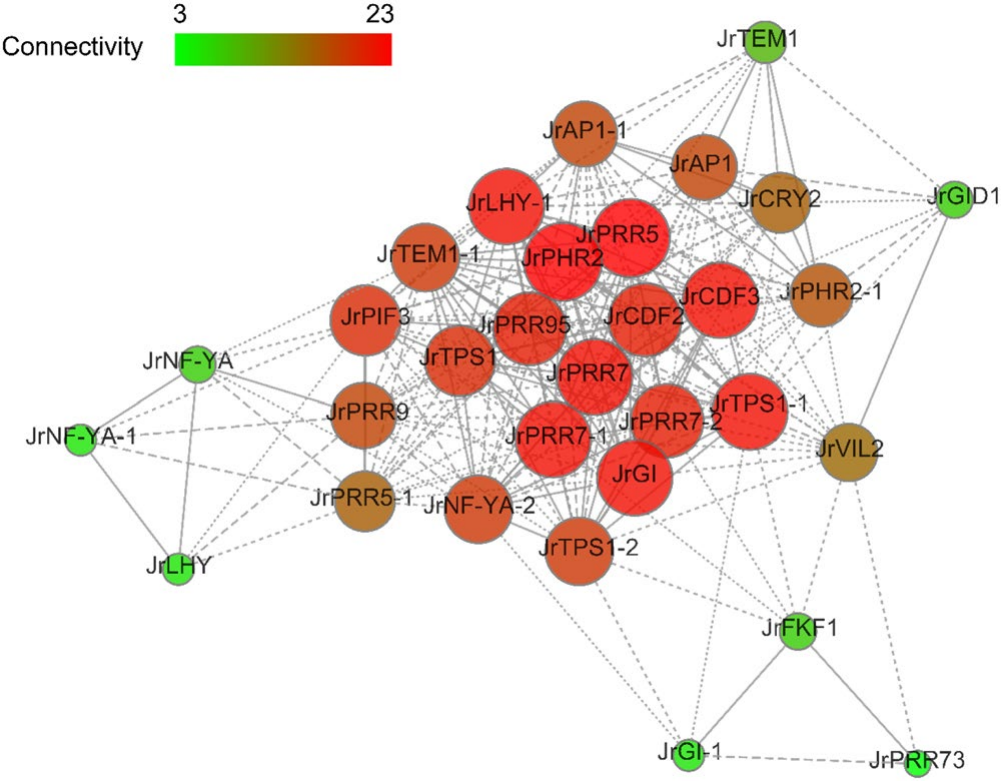
Coexpression networks of 31 DEGs related to flowering time. In the drawn weight network graph, the weight between genes is divided into four parts, which are represented by point lines, short dotted lines, long dotted lines and solid lines from light to heavy weights. Larger nodes and redder colors indicate greater connectivity of the genes in the network graph. The coexpression networks were drawn on the OmicShare website (http://www.omicshare.com/tools/Home/) based on the correlation coefficients between the genes.

Weighted gene coexpression network analysis (WGCNA) is a biology method for interaction analysis and resolving correlation networks^51^. To search for the genes involved in flowering time regulation in walnut, thirty-one flowering time-related DEGs were used to construct a coexpression network using the WGCNA method, and the results are presented in Fig. 11. In the coexpression network, many of the hub genes that participate in flowering time regulation were identified, such as *JrCDF-2*, *JrCDF-3*, *JrPRR7*, *JrPRR7-1*, *JrPRR7-2*, *JrTPS-1-1*, and the hub genes with the highest edge numbers were *JrCRY2* and *JrNF-YA-2*.

### Verification of DEG expression by qRT-PCR

**Figure 12:**
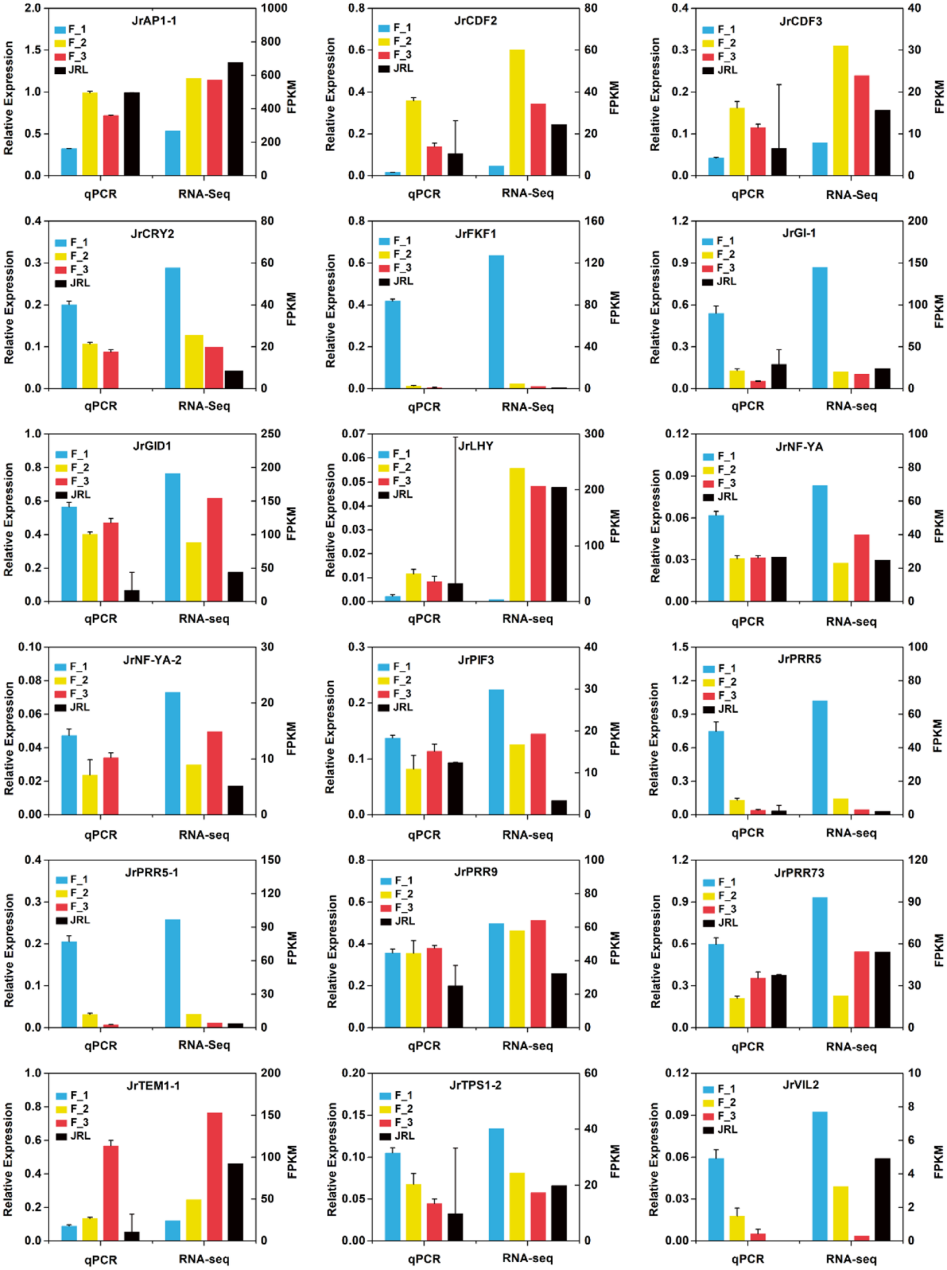
Verification of 18 flowering time-related DEGs by qRT-PCR.

To further verify the gene expression levels shown by RNA-Seq, we chose eighteen flowering time-related DEGs to perform qRT-PCR. Except for JrTPS1-2, the expression trends of the DEGs showed high similarity between the qPCR data and the RNA-Seq data (Fig. 12).

## Discussion

### Identification of the critical periods of female flower bud differentiation in walnut

Little is known about the development of walnut female flower buds, and initial differentiation is usually identified by a duration of one month after the female flowers bloom or four to six weeks after the medium and short twigs finish elongating. It is essential to ensure that experimental materials can be collected at precise times; however, due to regional differences in phenology, previous research conclusions cannot be used directly. In this experiment, we have preliminarily identified the initiation period of female flower bud differentiation in walnut through two years of observation of tissue sections, external morphological characteristics and local phenology. Generally, the stage of floral differentiation was divided into the physiological differentiation stage and morphological differentiation stage. Although we identified the initiation period of the morphological differentiation stage, the initiation period of the physiological differentiation stage and the DEGs involved in floral bud determination are still unknown in walnut and will be investigated in a future study. In addition, we suggest that some simple experimental methods, such as freehand sectioning or counting the number of bud scales can be used as preliminary judgment methods in field conditions (Fig. S1).

### Functional classification of genes

To functionally categorize the unigenes, the GO and KEGG databases were used to annotate the functions of the unigenes. GO enrichment showed that single organism process (GO:0044699, level 2, BP) was enriched, which included the GO subterm GO:0048449 (level 4) with the function of floral organ formation. The GO analysis of the DEGs indicated that the regulation of RNA metabolism, nucleic acid binding transcription factor activity and transcription factor activity were important during female flower bud differentiation. Interestingly, the GO terms of the DEGs between the flower buds (F_2) and leaf buds (JRL) were mainly enriched in functions related to photosynthesis. In total, 14 DEGs were associated with GO terms related to photosynthesis between F_2 and JRL, and the expression of these 14 DEGs were all upregulated in the leaf buds (Table S3), which suggested that the related genes upregulated in the leaf buds may participate in the process of carbohydrate assimilation. KEGG pathway analysis indicated that most pathways were associated with flowering and were shared by the four combinations of F_1vsF_2, F_1vsF_3, F_2vsF_3 and F_2vsJRL.

### Flowering pathways in walnut during the floral transition

Environmental cues (photoperiod, vernalization) and internal cues (autonomous, GA and age) result in the floral transition^3^. Most of the flowering time-related DEGs were enriched in the photoperiod pathway, and none of the DEGs were involved in the autonomous pathway or ambient temperature pathway. In the photoperiod pathway, the circadian rhythm genes, including *CCA1*, *FKF1*, *GI*, and *LHY*, can activate the photoperiodic hub gene *CO*. *CO* can activate *SOC1*, which can induce the expression of meristem identity genes, such as *LFY* and *AP1*, to activate the floral transition.

Interestingly, in the dimer of GI-FKF1 and trimer of CCA1-LHY-PIF3, the expression levels of the components in each complex were not consistent, such as *GI*, which showed a contrasting expression pattern to *FKF1*, as did *LHY* and *PIF3*. In addition, *PHR2* genes are annotated as blue-light photoreceptors, and CRY2 genes also function as blue-light receptors. They are flavoproteins similar in sequence and repressors of the CLOCK/BMAL1 heterodimer^29,52,53^. However, they showed contrasting expression patterns for reasons that remain unclear.

In the development of the floral transition, *PRRs*, *GI*, and *FKF1* were all downregulated from F_1 to F_2, while *LHY* and *CDFs* were upregulated from F_1 to F_2, suggesting that photoperiod pathway (circadian rhythm) genes may participate in the regulation of the floral transition.

In this study, the three stages of the female floral transition were identified by the morphological characteristics of tissue sections, and transcriptome-wide investigation of the gene expression profiles in walnut flower buds and leaf buds was conducted during the floral transition. Thirty-one DEGs related to flowering time were identified, and among them, *CRY2* and *NF-YA* were screened as core DEGs in flowering time regulation. Eighty-eight of the thirty-one DEGs (including *CRY2* and *NF-YA*) were confirmed by real-time quantitative analysis, and most of the RNA-Seq data were validated by qPCR data. Our work provides a foundation for further research on the walnut floral transition.

## Materials and Methods

### Plant materials

Walnut (*Juglans regia* L.) trees were grown under natural conditions in the southern part of the Xinjiang Uyghur Autonomous Region, China. Leaf buds were collected during the floral transition period (JRL), and female flower buds were collected before, during, after the floral transition period (F_1, F_2 and F_3). Each sample was pooled from 3 buds, and 3 biological repeats were performed, for a total of 9 buds for each stage of the floral transition. Three mixed samples from 9 buds were collected for sequencing, and the total RNA of each sample was extracted individually.

### Microscope observations

We peeled off the outer scales of the buds and fixed the buds in FAA fixative solution. Then, the fixed buds were dehydrated with a continuous gradient of ethanol and embedded in paraffin. Samples were cut into 8–12 μm slices (Leica Microtome, Germany), deparaffinized with xylene, and hydrated in a decreasing ethanol series. The sections were stained with Safranin and Fast Green and mounted with neutral gum. Finally, we observed the slices under a Motic microscope (Motic AE31, China).

### Transcriptome sequencing and library construction

Sequencing was carried out by the Novogene company, Beijing, China. Total RNA was extracted using RNAout 1.0 (Tianenze, Beijing, China). A total of 1.5 µg RNA per sample was used as input material for RNA sample preparation. Sequencing libraries were generated, and the clustering of the index-coded samples was performed. After cluster generation, the library preparations were sequenced on an Illumina HiSeq 2000 platform, and paired-end reads were generated.

### Quantitative real-time PCR

Total RNA was extracted using RNAout 1.0 (Tianenze, Beijing, China) by Novogene, Beijing, China. We synthesized the cDNA using the PrimeScript RT Reagent Kit (TaKaRa, Dalian, China). We performed real-time quantification using CFX Manager (Bio-Rad, USA) with SYBR Green Realtime PCR Master Mix (Toyobo, Osaka, Japan). The protocol of the real-time PCR was as follows: an initiation step at 95 °C for 5 min; followed by 40 cycles of 30 s at 94 °C, 30 s at 55 °C, and 30 s at 72 °C; and the melting curve step was processed from 65 to 95 °C. Each reaction was repeated for three times. The walnut Actin gene (forward primer: 5′-CCATCCAGGCTGTTCTCTC-3′, and reverse primer: 5′-GCAAGGTCCAGACGAAGG-3′) and walnut GAPDH gene (forward primer: 5′-ATTTGGAATCGTTGAGGGTCTTATG-3′ and reverse primer: 5′- AATGATGTTGAAGGAAGCAGCAC-3′) were used as the internal controls. The results were analyzed by the 2^−ΔΔCt^ method^54^.

### Differential expression analysis

For differential gene expression analysis, the read counts of each sequenced library were adjusted with EdgeR software. Differential expression analysis of two samples was performed using the DEGseq (2010) R package. The P values were adjusted using the q values^55^, and thresholds for significantly differential expression were set as q value < 0.005 and |log2(foldchange)| > 1.

## Supplementary information

Dataset 1

## References

[1] Pollegioni P (2017). Rethinking the history of common walnut (Juglans regia L.) in Europe: Its origins and human interactions. PLoS One.

[2] FAO, 2017, http://www.fao.org/faostat/en/#data/QC (2018).

[3] Khan MR, Ai XY, Zhang JZ (2014). Genetic regulation of flowering time in annual and perennial plants. Wiley Interdiscip Rev RNA.

[4] Bernier G, Havelange A, Houssa C, Petitjean A, Lejeune P (1993). Physiological Signals That Induce Flowering. Plant Cell.

[5] Simpson GG, Dean C (2000). Environmental-dependent acceleration of a developmental switch: the floral transition. Science’s STKE: signal transduction knowledge environment.

[6] Fornara F, de Montaigu A, Coupland G (2010). SnapShot: Control of flowering in Arabidopsis. Cell.

[7] Wigge PA (2005). Integration of spatial and temporal information during floral induction in Arabidopsis. Science.

[8] Hames C (2008). Structural basis for LEAFY floral switch function and similarity with helix-turn-helix proteins. Embo j.

[9] Irish VF (2010). The flowering of Arabidopsis flower development. Plant J.

[10] Chandler JW (2012). Floral meristem initiation and emergence in plants. Cell Mol Life Sci.

[11] Pose D, Yant L, Schmid M (2012). The end of innocence: flowering networks explode in complexity. Curr Opin Plant Biol.

[12] Torti S, Fornara F (2012). AGL24 acts in concert with SOC1 and FUL during Arabidopsis floral transition. Plant Signal Behav.

[13] Srikanth A, Schmid M (2011). Regulation of flowering time: all roads lead to Rome. Cell Mol Life Sci.

[14] Kardailsky I (1999). Activation tagging of the floral inducer FT. Science.

[15] Kobayashi Y, Kaya H, Goto K, Iwabuchi M, Araki T (1999). A pair of related genes with antagonistic roles in mediating flowering signals. Science.

[16] Abe M (2005). FD, a bZIP protein mediating signals from the floral pathway integrator FT at the shoot apex. Science.

[17] Corbesier L (2007). FT protein movement contributes to long-distance signaling in floral induction of Arabidopsis. Science.

[18] Moyroud E, Kusters E, Monniaux M, Koes R, Parcy F (2010). LEAFY blossoms. Trends in plant science.

[19] Roeder RG (1996). The role of general initiation factors in transcription by RNA polymerase II. Trends Biochem Sci.

[20] Nikolov DB, Burley SK (1997). RNA polymerase II transcription initiation: A structural view. Proceedings of the National Academy of Sciences of the United States of America.

[21] Lee TI, Young RA (2000). Transcription of eukaryotic protein-coding genes. Annual review of genetics.

[22] Brambilla V (2017). Antagonistic Transcription Factor Complexes Modulate the Floral Transition in Rice. Plant Cell.

[23] Goslin K (2017). Transcription Factor Interplay between LEAFY and APETALA1/CAULIFLOWER during Floral Initiation. Plant Physiol.

[24] Smaczniak C (2012). Characterization of MADS-domain transcription factor complexes in Arabidopsis flower development. Proc Natl Acad Sci USA.

[25] Simpson GG, Dean C (2002). Arabidopsis, the Rosetta stone of flowering time?. Science.

[26] Fornara F (2009). Arabidopsis DOF transcription factors act redundantly to reduce CONSTANS expression and are essential for a photoperiodic flowering response. Dev Cell.

[27] Imaizumi T, Schultz TF, Harmon FG, Ho LA, Kay SA (2005). FKF1 F-box protein mediates cyclic degradation of a repressor of CONSTANS in Arabidopsis. Science.

[28] Sawa M, Nusinow DA, Kay SA, Imaizumi T (2007). FKF1 and GIGANTEA complex formation is required for day-length measurement in Arabidopsis. Science.

[29] Kanai S, Kikuno R, Toh H, Ryo H, Todo T (1997). Molecular evolution of the photolyase-blue-light photoreceptor family. Journal of molecular evolution.

[30] Bagnall, D. J., King, R. W. & Hangarter, R. P. Blue-light promotion of flowering is absent in hy4 mutants of Arabidopsis. Planta 200, 278-280 (1996). 10.1007/BF00208319 8904811

[31] El-Din El-Assal S (2003). The role of cryptochrome 2 in flowering in Arabidopsis. Plant Physiol.

[32] Siriwardana CL (2016). NUCLEAR FACTOR Y, Subunit A (NF-YA) Proteins Positively Regulate Flowering and Act Through FLOWERING LOCUS T. PLoS Genet.

[33] Viczian A (2005). Functional characterization of phytochrome interacting factor 3 for the Arabidopsis thaliana circadian clockwork. Plant & cell physiology.

[34] Nakamichi N (2010). PSEUDO-RESPONSE REGULATORS 9, 7, and 5 are transcriptional repressors in the Arabidopsis circadian clock. Plant Cell.

[35] Castillejo C, Pelaz S (2008). The balance between CONSTANS and TEMPRANILLO activities determines FT expression to trigger flowering. Curr Biol.

[36] van Dijken AJ, Schluepmann H, Smeekens SC (2004). Arabidopsis trehalose-6-phosphate synthase 1 is essential for normal vegetative growth and transition to flowering. Plant Physiol.

[37] Wahl V (2013). Regulation of flowering by trehalose-6-phosphate signaling in Arabidopsis thaliana. Science.

[38] Chandler J, Wilson A, Dean C (1996). Arabidopsis mutants showing an altered response to vernalization. Plant J.

[39] Gendall AR, Levy YY, Wilson A, Dean C (2001). The VERNALIZATION 2 gene mediates the epigenetic regulation of vernalization in Arabidopsis. Cell.

[40] Levy YY, Mesnage S, Mylne JS, Gendall AR, Dean C (2002). Multiple roles of Arabidopsis VRN1 in vernalization and flowering time control. Science.

[41] Sung S, Amasino RM (2004). Vernalization in Arabidopsis thaliana is mediated by the PHD finger protein VIN3. Nature.

[42] Greb T (2007). The PHD finger protein VRN5 functions in the epigenetic silencing of Arabidopsis FLC. Curr Biol.

[43] Ratcliffe OJ, Nadzan GC, Reuber TL, Riechmann JL (2001). Regulation of flowering in Arabidopsis by an FLC homologue. Plant Physiol.

[44] Kim DH, Sung S (2010). The Plant Homeo Domain finger protein, VIN3-LIKE 2, is necessary for photoperiod-mediated epigenetic regulation of the floral repressor, MAF5. Proc Natl Acad Sci USA.

[45] Blazquez MA, Weigel D (2000). Integration of floral inductive signals in Arabidopsis. Nature.

[46] Eriksson S, Bohlenius H, Moritz T, Nilsson O (2006). GA4 is the active gibberellin in the regulation of LEAFY transcription and Arabidopsis floral initiation. Plant Cell.

[47] Alonso-Peral MM (2010). The microRNA159-regulated GAMYB-like genes inhibit growth and promote programmed cell death in Arabidopsis. Plant Physiol.

[48] Griffiths J (2006). Genetic characterization and functional analysis of the GID1 gibberellin receptors in Arabidopsis. Plant Cell.

[49] Harberd NP, Belfield E, Yasumura Y (2009). The angiosperm gibberellin-GID1-DELLA growth regulatory mechanism: how an “inhibitor of an inhibitor” enables flexible response to fluctuating environments. Plant Cell.

[50] Mandel MA, Gustafson-Brown C, Savidge B, Yanofsky MF (1992). Molecular characterization of the Arabidopsis floral homeotic gene APETALA1. Nature.

[51] Presson AP (2008). Integrated Weighted Gene Co-expression Network Analysis with an Application to Chronic Fatigue Syndrome. BMC Systems Biology.

[52] Biernat MA (2012). A baculovirus photolyase with DNA repair activity and circadian clock regulatory function. J Biol Rhythms.

[53] Cashmore AR, Jarillo JA, Wu YJ, Liu D (1999). Cryptochromes: blue light receptors for plants and animals. Science.

[54] Livak KJ, Schmittgen TD (2001). Analysis of relative gene expression data using real-time quantitative PCR and the 2(−Delta Delta C(T)) Method. Methods (San Diego, Calif.).

[55] Storey JD, Tibshirani R (2003). Statistical significance for genomewide studies. Proceedings of the National Academy of Sciences of the United States of America.

