# Supplementary material for: Stages identifying and transcriptome profiling of the floral transition in Juglans regia
Source: Sci Rep. 2019 May 8;9:7092. doi: 10.1038/s41598-019-43582-z (PMC6506622; doi:10.1038/s41598-019-43582-z)
Supplement: Supplementary file 1 — Dataset 1 [file 41598_2019_43582_MOESM1_ESM.docx]

Supplementary Information

**Stages identifying and transcriptome profiling of the** **floral transition in *Juglans regia***

**Shaowen Quan ^1, 2^, Jianxin Niu ^1, 2*^, Li Zhou ^1, 2^, Hang Xu^1, 2^, Li Ma^1, 2^, Yang Qin^1, 2^**

^1^ Department of Horticulture, College of Agriculture, Shihezi University, Shihezi 832003, Xinjiang, China.

^2^ Xinjiang Production and Construction Corps Key Laboratory of Special Fruits and Vegetables Cultivation Physiology and Germplasm Resources Utilization, Shihezi 832003, Xinjiang, China.

**^*^Correspondence:** Jianxin Niu

**Email:** njx105@163.com

Address: Department of Horticulture, College of Agriculture, Shihezi University, Shihezi 832003, Xinjiang, China.

Supplementary Information

**Figure S1.** Morphology diagram of walnut female flower buds.

**Figure S2.** The heatmap and the annotation of the flowering time related DEGs.

**Table S1.** The KEGG pathways associated with the walnut transcriptome data.

**Table S2.** Primers involved in this article.

**Table S3.** DEGs enriched under the GO terms of photosynthesis between F_2 and JRL.

**Figure S1.** Morphology diagram of walnut female flower buds.


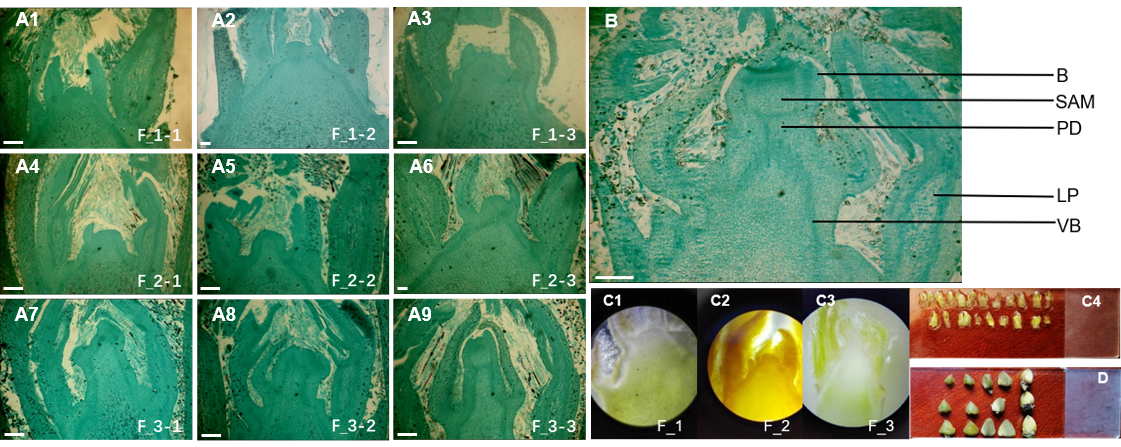


**A.** paraffin sections of walnut female flower buds under different differentiation periods, A1- A9 means the samples collected every three day, among them, A1 - A3 were the female flower buds identified in the undifferentiated stage (F_1), A4 - A6 were the female flower buds identified at the beginning of differentiation (F_2), A7 - A9 were the female flower buds identified at the flower primordia differentiation period(F_3); **B**. annotation of the walnut female flower bud, B means bracts; SAM means shoot apical meristem; PD means pedicel; LP means leaf primordium; VB means the vascular bundle (bars = 100 μm in a and b); **C**. free-hand sections of walnut female flower buds under different differentiation periods, C1 - C3 were the free-hand sections images of walnut female flower buds taken by mobile phone directly, C4 were the sliced materials; **D**. the scales and the left parts of the buds.

**Figure S2.** The heatmap and the annotation of the flowering time related DEGs.


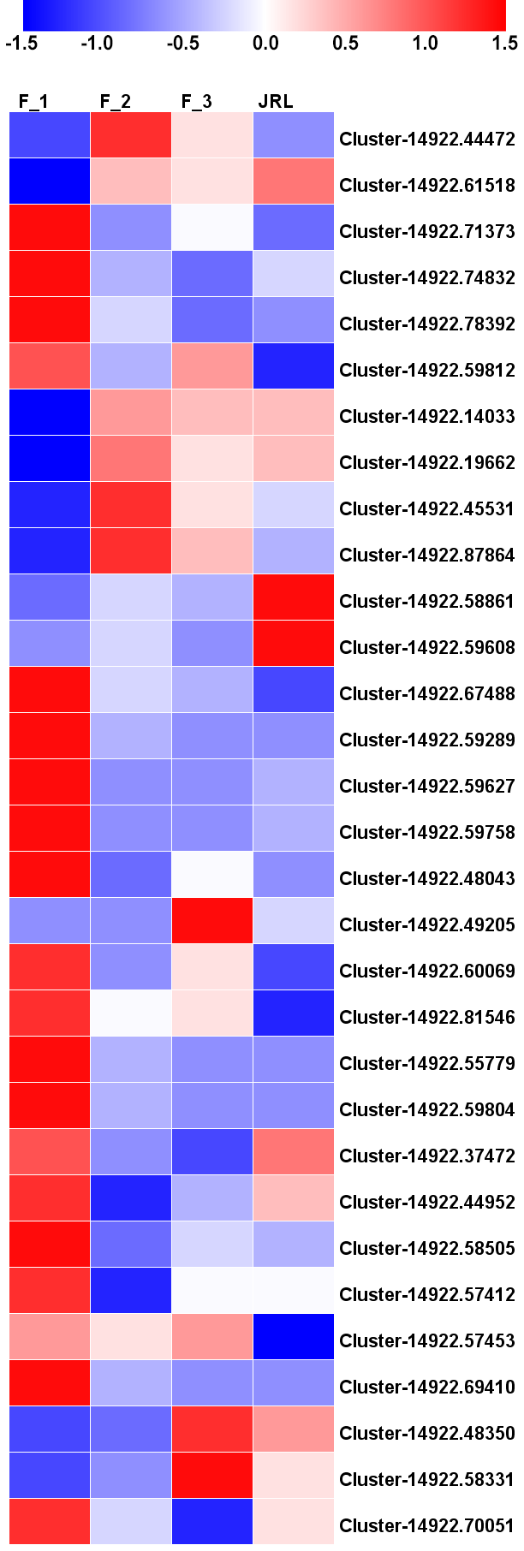


**Annotation**

Floral homeotic protein APETALA 1

Floral homeotic protein APETALA 1

Alpha,alpha-trehalose-phosphate synthase [UDP-forming]1

Alpha,alpha-trehalose-phosphate synthase [UDP-forming]1

Alpha,alpha-trehalose-phosphate synthase [UDP-forming]1

gibberellin receptor GID1

MYB-related transcription factor LHY

MYB-related transcription factor LHY

Cyclic dof factor 2

Cyclic dof factor 3

Blue-light photoreceptor PHR2

Blue-light photoreceptor PHR2

Cryptochrome-2

circadian clock-associated FKF1

GIGANTEA

GIGANTEA

CCAAT-binding transcription factor (CBF-B/NF-YA) subunit B

CCAAT-binding transcription factor (CBF-B/NF-YA) subunit B

CCAAT-binding transcription factor (CBF-B/NF-YA) subunit B

Phytochrome interacting factor 3

pseudo-response regulator 5

pseudo-response regulator 5

pseudo-response regulator 7 (PRR7)

pseudo-response regulator 7 (PRR7)

pseudo-response regulator 7 (PRR7)

Two-component response regulator-like PRR73

pseudo-response regulator 9 (PRR9)

Two-component response regulator-like PRR95

AP2/ERF and B3 domain-containing transcription repressor TEM1

AP2/ERF and B3 domain-containing transcription repressor TEM1

VIN3-like protein 2

**Gene**

AP1(F)

AP1-1(F)

TPS1(F)

TPS1-1(F)

TPS1-2(F)

GID1(F)

LHY(F)

LHY-1(F)

CDF2(F&FL)

CDF3(F)

PHR2 (FL)

PHR2-1(FL)

CRY2(F&FL)

FKF1(F)

GI(F)

GI-1(F)

NF-YA(F)

NF-YA-1(F)

NF-YA-2(F)

PIF3(FL)

PPR5(F)

PPR5-1(F)

PRR7(F)

PRR7-1(F)

PRR7-2(F)

PRR73(F)

PRR9(FL)

PRR95(F)

TEM1(F&FL)

TEM1-1(F)

VIL2(F)

**Pathway**

supplyment

supplyment

Age regulated

Age regulated

Age regulated

GA

Integrators

Integrators

Photoperiod

Photoperiod

Photoperiod

Photoperiod

Photoperiod

Photoperiod

Photoperiod

Photoperiod

Photoperiod

Photoperiod

Photoperiod

Photoperiod

Photoperiod

Photoperiod

Photoperiod

Photoperiod

Photoperiod

Photoperiod

Photoperiod

Photoperiod

Photoperiod

Photoperiod

Vernalization

| Pathway Hierarchy1 | Pathway Hierarchy2 | KEGG Pathway Description | Pathway ID | Gene Number |
| --- | --- | --- | --- | --- |
| Cellular Processes | Transport and catabolism | Endocytosis; Peroxisome; Phagosome; Regulation of autophagy | ko04144; ko04146 ko04145; ko04140 | 1200 |
| **Environmental Information Processing** | Membrane transport | ABC transporters | ko02010 | 147 |
|  | **Signal transduction** | Phosphatidylinositol signaling system; **Plant hormone signal transduction** | ko04070; **ko04075** | 900 |
| Genetic Information Processing | Folding, sorting and degradation | Proteasome; Protein export; Protein processing in endoplasmic reticulum; RNA degradation; SNARE interactions in vesicular transport; Sulfur relay system; Ubiquitin mediated proteolysis | ko03050; ko03060; ko04141; ko03018; ko04130; ko04122; ko04120 | 1792 |
|  | Replication and repair | Base excision repair; DNA replication; Homologous recombination; Mismatch repair; Non-homologous end-joining; Nucleotide excision repair | ko03410; ko03030; ko03440; ko03430; ko03450; ko03420 | 553 |
|  | Transcription | Basal transcription factors; RNA polymerase; Spliceosome; | ko03022; ko03020; ko03040 | 877 |
|  | Translation | Aminoacyl-tRNA biosynthesis; RNA transport; Ribosome;  Ribosome biogenesis in eukaryotes; mRNA surveillance pathway | ko00970; ko03013; ko03010; ko03008; ko03015 | 2170 |
| Metabolism | Amino acid metabolism | Alanine; aspartate and glutamate metabolism; Arginine and proline metabolism; Arginine biosynthesis; Cysteine and methionine metabolism; Glycine; serine and threonine metabolism Histidine metabolism; Lysine biosynthesis; Lysine degradation; Phenylalanine metabolism; Phenylalanine; tyrosine and tryptophan biosynthesis; Tryptophan metabolism; Tyrosine metabolism; Valine; leucine and isoleucine biosynthesis Valine; leucine and isoleucine degradation | ko00250; ko00330; ko00220; ko00270; ko00260; ko00340; ko00300; ko00310; ko00360; ko00400; ko00380; ko00350; ko00290; ko00280 | 1428 |
|  | Biosynthesis of other secondary metabolites | Anthocyanin biosynthesis; Betalain biosynthesis; Caffeine metabolism; Flavone and flavonol biosynthesis; Flavonoid biosynthesis; Glucosinolate biosynthesis; Isoflavonoid biosynthesis; Isoquinoline alkaloid biosynthesis; Monobactam biosynthesis; Phenylpropanoid biosynthesis; Stilbenoid; diarylheptanoid and gingerol biosynthesis; Tropane; piperidine and pyridine alkaloid biosynthesis | ko00942; ko00965; ko00232; ko00944; ko00941; ko00966; ko00943; ko00950; ko00261; ko00940; ko00945; ko00960 | 574 |
|  | Carbohydrate metabolism | Amino sugar and nucleotide sugar metabolism; Ascorbate and aldarate metabolism; Butanoate metabolism; C5-Branched dibasic acid metabolism; Citrate cycle (TCA cycle); Fructose and mannose metabolism; Galactose metabolism; Glycolysis / Gluconeogenesis; Glyoxylate and dicarboxylate metabolism; Inositol phosphate metabolism; Pentose and glucuronate interconversions; Pentose phosphate pathway; Propanoate metabolism; Pyruvate metabolism; Starch and sucrose metabolism | ko00520; ko00053; ko00650; ko00660; ko00020; ko00051; ko00052; ko00010; ko00630; ko00562; ko00040; ko00030; ko00640; ko00620; ko00500 | 2152 |
|  | Energy metabolism | Carbon fixation in photosynthetic organisms; Nitrogen metabolism; Oxidative phosphorylation; Photosynthesis; Photosynthesis - antenna proteins; Sulfur metabolism | ko00710; ko00910; ko00190; ko00195; ko00196; ko00920 | 1113 |
|  | Glycan biosynthesis and metabolism | Glycosaminoglycan degradation; Glycosphingolipid biosynthesis - ganglio series; Glycosphingolipid biosynthesis-globo series; Glycosylphos phatidyl inositol(GPI)-anchor biosynthesis; N-Glycan biosynthesis; Other glycan degradation; Other types of O-glycan biosynthesis | ko00531; ko00604; ko00603; ko00563; ko00510; ko00511; ko00514 | 412 |
|  | Lipid metabolism | Arachidonic acid metabolism; Biosynthesis of unsaturated fatty acids; Cutin; suberine and wax biosynthesis; Ether lipid metabolism; Fatty acid biosynthesis; Fatty acid degradation; Fatty acid elongation; Glycerolipid metabolism; Glycerophospholipid metabolism; Linoleic acid metabolism; Sphingolipid metabolism; Steroid biosynthesis; Synthesis and degradation of ketone bodies; alpha-Linolenic acid metabolism | ko00590; ko01040; ko00073; ko00565; ko00061; ko00071; ko00062; ko00561; ko00564; ko00591; ko00600; ko00100; ko00072; ko00592 | 1255 |
|  | Metabolism of cofactors and vitamins | Biotin metabolism; Folate biosynthesis; Lipoic acid metabolism; Nicotinate and nicotinamide metabolism; One carbon pool by folate; Pantothenate and CoA biosynthesis; Porphyrin and chlorophyll metabolism; Riboflavin metabolism; Thiamine metabolism; Ubiquinone and other terpenoid-quinone biosynthesis; Vitamin B6 metabolism | ko00780; ko00790; ko00785; ko00760; ko00670; ko00770; ko00860; ko00740; ko00730; ko00130; ko00750 | 941 |
|  | Metabolism of other amino acids | Cyanoamino acid metabolism; Glutathione metabolism; Selenocompound metabolism; Taurine and hypotaurine metabolism; beta-Alanine metabolism | ko00460; ko00480; ko00450; ko00430; ko00410 | 680 |
|  | Metabolism of terpenoids and polyketides | Brassinosteroid biosynthesis; Carotenoid biosynthesis; Diterpenoid biosynthesis; Limonene and pinene degradation; Monoterpenoid biosynthesis; Sesquiterpenoid and triterpenoid biosynthesis; Terpenoid backbone biosynthesis; Zeatin biosynthesis | ko00905; ko00906; ko00904; ko00903; ko00902; ko00909; ko00900; ko00908 | 551 |
|  | Nucleotide metabolism | Purine metabolism; Pyrimidine metabolism | ko00230; ko00240 | 834 |
|  | Overview | 2-Oxocarboxylic acid metabolism; Biosynthesis of amino acids; Carbon metabolism; Degradation of aromatic compounds; Fatty acid metabolism | ko01210; ko01230; ko01200; ko01220; ko01212 | 1575 |
| **Organismal Systems** | **Environmental adaptation** | **Circadian rhythm - plant;** Plant-pathogen interaction | ko04712; ko04626 | 1047 |

**Table S1.** The KEGG pathways associated with the walnut transcriptome data.

| **Gene** | Forward primer (5’-3’) | Reverse primer (5’-3’) |
| --- | --- | --- |
| JrAP1-1 | GCTGGTCATCATCAAGGAA | GGCTGAGGAGGTTGGTAT |
| JrCDF2 | CCGAGGTTGGTTGACATT | TGAGAAGGAGAGTGCTACA |
| JrCDF3 | ACTCTGCCTCACATTATCG | GTCCATCACCTGCTTCTC |
| JrCRY2 | GAGACATACTGCCAACCAA | CCATCGGACTTAGAGAATACT |
| JrFKF1 | ACCACGAATCATATCCAGTT | GCATACCATCTTCCTCACA |
| JrGI-1 | AACCGTAGGCACTTCTCT | CTCTGGCATTAGGCTTCC |
| JrGID1 | CTCCAGACCAAGAACAACT | TCACCTACAGCAGCCATA |
| JrLHY | CAGCAACAATGGAGAGGAT | TCGCAGACAATAGACATACC |
| JrNF-YA | AGTTATGCCTGCTTATGGAT | TTGGAGTATGTTGGTTGTTG |
| JrNF-YA-2 | TACCGTTACCATTGTTGCTA | GTTCCACTGCCACTTGAT |
| JrPIF3 | AAGTGCCGAGTTGATACG | TGCGACCTAAGAGACCTAA |
| JrPRR5 | AAGGAGTCACCATCAGTTC | GGAGGACCGAGGACATAT |
| JrPRR5-1 | AGCACTTCCATTCACACTAT | CCACCACCACCATTATCAT |
| JrPRR9 | TGTCCAAGTTCCTCCATCT | CCTCAGTTCAATCCAATCCT |
| JrPRR73 | CCACTTGAACGGTTGCTA | TGCTGCTGACTATCTTGTTA |
| JrTEM1-1 | GTCCTCCTCTTCAGTCGTA | CACAACCAGCACAATCTTAG |
| JrTPS1-2 | CCGTCTTCATAGTGCTTGT | CTCTTGGTGGTGGCTAAC |
| JrVIL2 | CCTTCAGAACCTACTCGTAA | TCAGAGCATCATCACATCC |
| JrACTIN | GCCGAACGGGAAATTGTC | AGAGATGGCTGGAAGAGG |
| JrGADPH | ATTTGGAATCGTTGAGGGTCTTATG | AATGATGTTGAAGGAAGCAGCAC |

**Table S2.** Primers involved in this article.

| gene_id | sample_  fpkm  (F_2) | sample_  fpkm  (JRL) | UP_DOWN_  REGULATION  (F_2vsJRL) | log2ratio  (F_2vsJRL) | q value  (F_2vsJRL) | Gene Ontology  Biological Pathway | BP Description |
| --- | --- | --- | --- | --- | --- | --- | --- |
| Cluster-14922.37469 | 12.72 | 27.78 | DOWN | -1.1523 | 0.00397 | GO:0015979 | photosynthesis |
| Cluster-14922.43588 | 77.29 | 185.2 | DOWN | -1.284 | 1.1E-05 | GO:0015979 | photosynthesis |
| Cluster-14922.48757 | 64.97 | 145.07 | DOWN | -1.1833 | 9.7E-07 | GO:0015979 | photosynthesis |
| Cluster-14922.51652 | 93.33 | 255.86 | DOWN | -1.4784 | 5.1E-11 | GO:0015979 | photosynthesis |
| Cluster-14922.51968 | 78.08 | 198.89 | DOWN | -1.3734 | 1.5E-13 | GO:0015979 | photosynthesis |
| Cluster-14922.53551 | 35.4 | 126.76 | DOWN | -1.8643 | 1.4E-10 | GO:0015979 | photosynthesis |
| Cluster-14922.54865 | 81.08 | 274.45 | DOWN | -1.7821 | 3.2E-13 | GO:0015979 | photosynthesis |
| Cluster-14922.56766 | 85.95 | 223.85 | DOWN | -1.4052 | 1.8E-13 | GO:0009765 | photosynthesis, light harvesting |
| Cluster-14922.58912 | 192.95 | 393.65 | DOWN | -1.0512 | 1.1E-06 | GO:0015979 | photosynthesis |
| Cluster-14922.59270 | 227.38 | 583.37 | DOWN | -1.3831 | 1.1E-27 | GO:0015979  //GO:0030494 | Photosynthesis  //bacteriochlorophyll biosynthetic process |
| Cluster-14922.60136 | 30.03 | 77.65 | DOWN | -1.4502 | 2.2E-09 | GO:0015979 | photosynthesis |
| Cluster-14922.65853 | 45.64 | 170.13 | DOWN | -1.9204 | 1.6E-06 | GO:0015979  //GO:0006418 | Photosynthesis  //tRNA aminoacylation for protein translation |
| Cluster-14922.67771 | 50.91 | 196.87 | DOWN | -1.9746 | 2.4E-12 | GO:0015979 | photosynthesis |
| Cluster-14922.76533 | 66.25 | 138.23 | DOWN | -1.0852 | 0.00017 | GO:0015979 | photosynthesis |

**Table S3.** DEGs enriched under the GO terms of photosynthesis between F_2 and JRL.
